# Motor module activation sequence and topography in the spinal cord during air‐stepping in human: Insights into the traveling wave in spinal locomotor circuits

**DOI:** 10.14814/phy2.13504

**Published:** 2017-11-28

**Authors:** Hikaru Yokoyama, Kohtaroh Hagio, Tetsuya Ogawa, Kimitaka Nakazawa

**Affiliations:** ^1^ Laboratory of Sports Sciences Department of Life Sciences Graduate School of Arts and Sciences The University of Tokyo Tokyo Japan; ^2^ Japan Society for the Promotion of Science Chiyoda‐ku, Tokyo Japan

**Keywords:** Central pattern generator, Locomotion, Locomotor module, Muscle synergy

## Abstract

Coordinated locomotor muscle activity is generated by the spinal central pattern generators (CPGs), which are modulated by peripheral and supraspinal inputs. The CPGs would consist of multiple motor modules generating basic muscle activity, which are distributed rostrocaudally along the spinal cord. To activate the motor modules in proper sequence, rostrocaudally traveling waves of activation in the spinal cord are important mechanisms in the CPGs. The traveling waves of activation have been observed in nonhuman vertebrates. However, they have not yet been confirmed during human locomotion. Although, rostrocaudal wave‐like activations in the spinal cord were observed during walking in humans in a previous study, the propagation shifted rostrally toward the upper lumbar segments at foot contact. Here, using an air stepping task to remove the foot‐contact interactions, we examined whether the traveling wave mechanism exists in the human spinal circuits based on the activation sequence of motor modules and their topography. We measured electromyographic activity of lower leg muscles during the air‐stepping task. Then, we extracted motor modules (i.e., basic patterns of sets of muscle activations: muscle synergies) from the measured muscle activities using nonnegative matrix factorization method. Next, we reconstructed motoneuron (MN) activity from each module activity based on myotomal charts. We identified four types of motor modules from muscle activities during the air‐stepping task. Each motor module represented different sets of synergistic muscle activations. MN clusters innervating each motor module were sequentially activated from the rostral to caudal region in the spinal cord, from the initial flexion to the last extension phase during air‐stepping. The rostrocaudally sequential activation of MN clusters suggests the possibility that rostrocaudally traveling waves exist in human locomotor spinal circuits. The present results advance the understanding of human locomotor control mechanisms, and provide important insights into the evolution of locomotor networks in vertebrates.

## Introduction

The timing and pattern of locomotor muscle activities in vertebrates are generated by spinal neural networks referred to as spinal central pattern generators (CPGs) (Grillner 1981; Kiehn 2016). Recent animal studies combining electrophysiology with molecular genetics demonstrated that the CPGs consisted of multiple types of spinal interneurons (Kiehn 2016). In humans, indirect evidence of the existence of CPGs has been demonstrated by several studies in patients with spinal cord injury (SCI) (Calancie et al. 1994; Dimitrijevic et al. 1998; Danner et al. 2015).

Regarding spinal motor control, the anatomical positions of the motoneurons (MNs), which receive inputs from CPGs, are logically arranged according to the biomechanical characteristics of their target muscles (Romanes 1964; Jessell et al. 2011). Generally, each muscle is innervated from several spinal segments and each spinal segment innervates several muscles. The anatomical grouping at each segment may reflect synergistic functions at a given hind limb joint along the rostrocaudal axis of the spinal cord (Romanes 1964). In addition, MN columns that innervate antagonist muscles are separated spatially along the mediolateral axis of the spinal cord (McHanwell and Biscoe 1981). Recently, such stereotyped organization of MNs was suggested to simplify the connectivity between MNs and premotor inputs for locomotor control in the mouse (Hinckley et al. 2015). In human bipedal walking, specificity in the MN arrangement along the mediolateral axis depending on gait phase (i.e., stance and swing phase) has been suggested (Ivanenko et al. 2008).

Regarding the control of locomotor muscle activity, a small number of motor modules (also referred as muscle synergies) generate complex activities of various muscles (Tresch et al. 1999). The motor modules are encoded in spatial pattern formation networks, which activate multiple MN pools, in the spinal CPGs (McCrea and Rybak 2008). The CPGs are considered to consist of the pattern formation networks and temporal regulation networks, which send activation commands to the pattern formation networks (McCrea and Rybak 2008). A recent study by Saltiel et al. (2015) demonstrated that a focal neurochemical stimulation to the frog spinal cord elicited specific types of motor module activities similar to those used in intact frog locomotion. Interestingly, the locomotor modules exhibited partially overlapping representations along the rostrocaudal direction. Further, the order from rostral to caudal segments corresponded to the activation sequence in a gait cycle. The relationships between the activation sequence of locomotor muscle synergies and their spinal cord topography suggests that the locomotor muscle activity is generated by a wave of neural activation, traveling in the rostrocaudal direction, in the lumbosacral spinal cord. The traveling wave is assumed to be derived from rostrocaudal propagation of electrical activity of dorsal horn neurons and relevant to the temporal regulation networks of CPGs (Cuellar et al. 2009). A simulation model demonstrated that sequentially spacing motor modules from the rostral to caudal regions and the rostrocaudal traveling wave, acted as the pattern formation networks and the temporal regulation networks, respectively, to reproduce actual motor outputs in frogs (Saltiel et al. 2015).

The traveling wave of motor output has been observed among different vertebrates, especially in animals using undulating locomotion, including Lamprey (Wallén and Williams 1984), fish (Grillner 1974), and tadpoles (Roberts et al. 1998). Although there is still debate about whether the traveling wave mechanisms exist in legged animals (Cuellar et al. 2009; Pérez et al. 2009; Auyong et al. 2011), evidence for its existence has been observed in frogs (Saltiel et al. 2015), rodents (Cazalets 2005), and cats (Cuellar et al. 2009; Pérez et al. 2009). However, thus far, the traveling wave has not yet been confirmed in human locomotion. Based on myotomal charts (Kendall et al. 1993), a previous study reconstructed the MN activation during walking in humans (Ivanenko et al. 2006). They demonstrated the rostrocaudal movement of MN activations in the lumbosacral enlargement during a gait cycle based on the locus of the center of activity (CoA) of the MN activity Although the CoA demonstrated the rostrocaudally traveling wave‐like activation of MNs from upper lumbar to lower sacral segments in a swing‐stance cycle, the CoA shifted rostrally toward the upper lumbar segments at foot contact in the middle of the rostrocaudal propagation. The rostral shift of the CoA was induced by a motor module activating the quadriceps and tibialis anterior (TA) at foot contact related to loading response (Ivanenko et al. 2006). A comparative study between humans walking and animal locomotion demonstrated that the large activity of quadriceps and TA at foot contact related to loading response is unique to human upright walking (Vilensky 1987).

Generally, a large part of the spinal CPGs mechanisms is phylogenetically conserved across different species (Goulding 2009; Yokoyama et al. 2017). Based on the commonality of the locomotor circuits, it is quite possible that the traveling wave of activation exists in the human spinal circuits. The traveling wave of activation in the spinal cord may not have been observed in the previous human walking study (Ivanenko et al. 2006) due to it being masked by the muscle activity induced by loading response at foot contact The air‐stepping task has been previously used to examine human locomotor control under the conditions without foot‐contact interactions, thus, eliminating body‐weight loading (Ivanenko et al. 2002, 2007). By removing foot‐contact interactions, the motor output of air‐stepping might represent more endogenous activity in the spinal CPGs compared with that during normal walking. Thus, in this study, by adopting an air‐stepping task we examined whether the traveling wave of activation exists in the human spinal circuits based on activation sequence of motor modules and their innervation locations in the lumbosacral enlargement during air stepping. Here, assuming that the traveling wave of activation exists in the human spinal circuits and recruits motor modules during air‐stepping, we hypothesized that motor modules would be sequentially recruited in the order the innervation locations from rostral to caudal regions during each step. The acceptance of this working hypothesis would provide indirect evidence that the traveling wave mechanisms are conserved in humans. Our results would contribute to better understanding the human locomotor system and commonality of locomotor neural systems among vertebrates.

## Materials and Methods

### Participants

Nine healthy male volunteers (age ± standard deviation [SD], 26.1 ± 2.7 years) participated in this study. Each participant gave written informed consent for his participation in the study. This study was carried out in accordance with the Declaration of Helsinki and with the approval of the Ethics Committee of the Graduate School of Arts and Sciences, the University of Tokyo.

### Experimental setup and design

**Figure 1:**
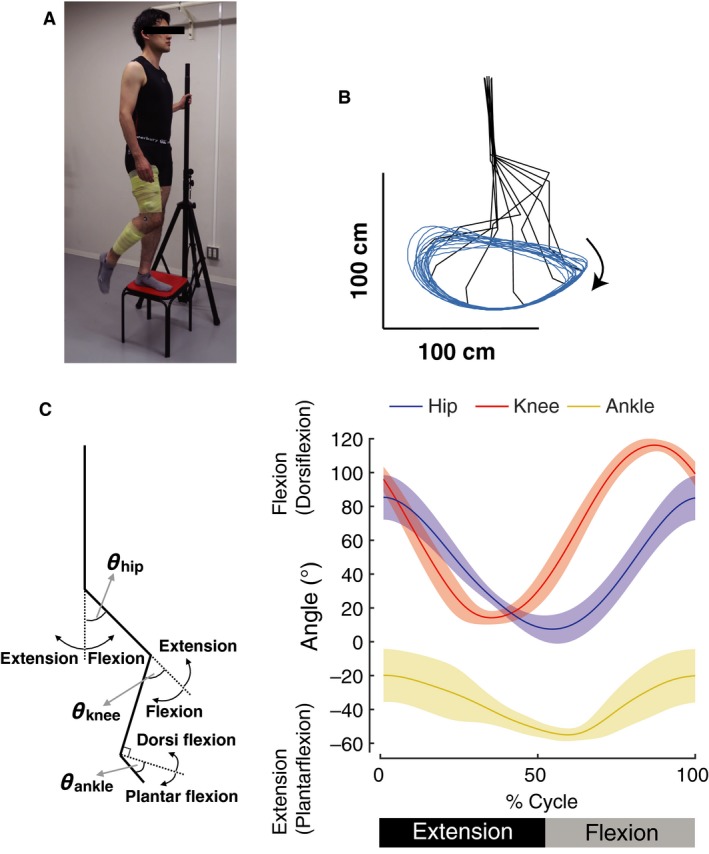
(A) Experimental setup of one‐leg air stepping movement. Participants stepped with the right leg in the air while standing on a stool on the left leg and holding a vertical pole with the left hand for stabilization. In accordance with the research ethics of the journal, the individual gave written consent for the publication of this image. (B) An example of kinematics of one‐leg air stepping movement. Averaged kinematics patterns over 20 consecutive step cycles are shown at every 10% gait cycle for a single participant. Blue line indicates the trajectory of the toe marker over the 20 consecutive step cycles. (C) Ensemble averages across participants (± standard deviation) of the hip, knee, and ankle angles in an extension‐flexion cycle based on the hip angle.

Participants stepped with the right leg in the air while standing on a stool on the left leg and holding a vertical pole with the left hand for stabilization (Fig. 1A). They were instructed to perform 30 strides of one‐leg air‐stepping at a comfortable cadence as if they walked on the ground. In this condition, all participants performed the step at a pace of 1.34 ± 0.12 sec/step (mean ± SD). The step frequency was approximately equivalent to the stride time of slow walking (1.38 sec/stride at 0.83 m/sec) (Murray et al. 1984). Prior to the experiment, the participants practiced the task for 5 min.

### Data collection

Surface electromyogram (EMG) was recorded from the following 14 muscles on the right leg: TA, gastrocnemius medialis (MG), soleus (SOL), peroneus longus (PL), vastus lateralis (VL), rectus femoris (RF), biceps femoris long head (BF), semitendinosus (ST), adductor longus (AL), sartorius (SART), iliopsoas (ILIO), tensor fascia latae (TFL), gluteus maximus (GM), and gluteus medius (Gmed). Electrodes were placed in accordance with the recommendation of Criswell and Cram (2011). Although the ILIO is a deep muscle, the superficial area of the ILIO is adequately large for surface EMG recording (Jiroumaru et al., 2014). Nevertheless, EMG signals of the ILIO can be corrupted by cross talk from adjacent hip flexors. Thus, to minimize cross talk from adjacent muscles, we carefully checked location of the ILIO by manual palpation as outlined by Muscolino (2008) and performed cross‐talk tests suggested by Criswell and Cram (2011). The EMG was recorded with a wireless EMG system (Trigno Wireless System; DELSYS, Boston, MA, USA). The EMG signals were band‐pass filtered (20–450 Hz) and sampled at 1,000 Hz with the EMG system and a multichannel data‐recording unit (PowerLab System, AD Instruments, Sydney, Australia), respectively. Kinematic data were recorded at 100 Hz by using an optical motion capture system (OptiTrack: V100R2, Natural Point, OR, USA) with six cameras. Five spherical markers were placed over the right side of the fifth metatarsal head (toe), lateral malleolus (ankle), lateral femoral epicondyle (knee), greater trochanter (hip), and acromion process (shoulder).

### Kinematic analysis

The kinematic signals were digitally smoothed with a zero‐lag low‐pass Butterworth filter (6‐Hz cutoff, fourth order). From the marker coordinates, the joint angles at the ankle, knee, and hip were calculated (Definition: Fig. 1C, stick diagram). The beginnings of step cycles were defined as the peak flexion timing of the hip joint angle. Of the recorded 30‐step cycles, we used 20‐cycle data (EMG and kinematics signals) excluding the first and the last five cycles for subsequent analysis.

### EMG processing

The EMG data were demeaned, rectified, and smoothed with a zero‐lag low‐pass Butterworth filter (6‐Hz cutoff, fourth order) to obtain the EMG envelopes (Walter et al. 2014). Subsequently, the processed EMG data were time‐interpolated so that they had 200 points for each gait cycle.

### Extraction of motor modules from the EMG data

**Figure 2:**
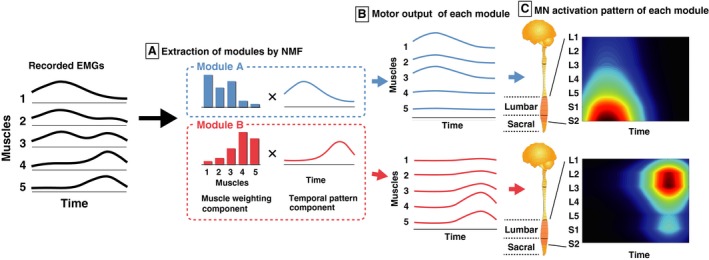
Procedures of the reconstruction of the spatio‐temporal activation patterns of motoneurons (MNs) along the rostrocaudal axis of the lumbosacral enlargement for individual motor modules from the EMGs. (A) Motor modules were extracted using nonnegative matrix factorization (NMF) from EMGs. (B) The output of each module is explained by the product of the muscle weighting component (bars: specifying activation level of each muscle) and the temporal pattern component (waveforms). The sum of outputs from the modules is approximately equivalent to that of the EMGs. (C) The MN activity in each segment (L2–S2, left vertical scale) generated from each module was reconstructed by mapping the output of each module based on Kendall's myotomal charts (Table 1).

Motor modules were extracted from the processed EMG data using the nonnegative matrix factorization (NMF, Fig. 2A) (Lee and Seung 1999; Dominici et al. 2011). Motor modules were extracted in each participant from an EMG dataset organized as a matrix with 14 muscles × 4000 variables (i.e., 20‐step cycles × 200 time points). By using the NMF, the EMG data matrix, ***M***, was decomposed into spatial muscle weightings, ***W***, which correspond to the motor modules, and their temporal activations, ***C***, according to equation (1):

(1) M=W·C+e

where ***M*** (***m *** ×  ***t*** matrix, where ***m*** is the number of muscles and ***t*** is the number of time points, the EMG data matrix) is a linear combination of motor modules, ***W*** (***m *** ×  ***N***_***module***_ matrix, where ***N***_***module***_ is the number of motor modules), and their temporal activation patterns, ***C*** (***N***_***module***_ × ***t*** matrix), and ***e*** is the residual error. The optimal number of motor modules ***N***_***module***_ was determined by iterating each possible ***N***_***module***_ from 1 to 10. For each ***N***_***module***_, the goodness of fit was evaluated based on the variance accounted for (VAF) (Torres‐Oviedo et al. 2006). Based on the VAF, the optimal module number ***N***_***module***_ was defined as the minimum number fulfilling the following three criteria: (1) number of modules achieving VAF > 90% (Torres‐Oviedo et al. 2006), (2) number to which adding an additional module did not increase VAF by > 5% of VAF (Frere and Hug 2012), and (3) number of modules selected based on the “cusp” method proposed by Cheung et al. (2009). This method selects the smallest ***N***_***module***_ such that the increase in VAF resulting from an additional module is lower than the increase of 75% of the almost linear slope of VAF calculated for a randomized data matrix of EMG (i.e., the number beyond which any further increase in the number of extracted modules yields a VAF increase < 75% of that expected from random chance) (for details see (Cheung et al. 2009; Yokoyama et al. 2016).

After the extraction of motor modules, we clustered the extracted motor modules using hierarchical clustering analysis to examine their types (Ward's method, correlation distance) based on the muscle weightings, as in our previous study (Yokoyama et al. 2016, 2017). The clustering analysis was performed for all participants of all motor modules. The optimal cluster number was selected by the gap statistic (Tibshirani et al. 2001).

### Spatiotemporal activation patterns of MNs within the spinal cord generated by each motor module

Based on the muscle activity generated from each module (i.e., the product of the muscle weightings and the corresponding temporal activation, Fig. 2B), spatiotemporal activation patterns of MNs were reconstructed from individual modules of all participants (Fig. 2C). The muscle activity generated from each module was mapped onto the estimated rostrocaudal location of the MN pools in the spinal cord from the L1 to S2 segments. We used Kendall's myotomal charts (Kendall et al. 1993) as in previous studies (Ivanenko et al. 2006; Yokoyama et al. 2017). In the myotomal charts, the weight coefficients of the innervation level are expressed as ***x ***=*** ***0.5 or ***X ***=*** ***1.0 (Table 1). Based on the weightings, the MN activity patterns of the ***j***th spinal segment ***Sj*** was estimated according to the following equation (2),

(2) $$S_{j}=\frac{\sum_{i=1}^{n_{j}}k_{\mathrm{ij}}\cdot \mathrm{Muscle}\;\mathrm{activit}y_{i}}{n_{j}},$$

where ***Muscle activity***_***i***_ is the ***i***th muscle activity generated from a motor module, ***nj*** is the number of ***Muscle activity***_***i***_ corresponding to the ***j***th segment and ***k***_***ij***_ takes a value either ***x*** or ***X*** as a weighting coefficient of the ***i***th muscle for the ***j***th spinal segment. To visualize a smooth spatiotemporal activation in the rostrocaudal segments of the spinal cord, we used a filled contour plot (Ivanenko et al. 2006; Yokoyama et al. 2017).

**Table 1:** Muscle innervation charts. Data are adopted from Kendall et al. (1993)

	ILIO	GM	Gmed	TFL	SART	AL	RF	VL	BF	ST	MG	SOL	PL	TA
L1	x													
L2	X				X	X	X	X						
L3	X				X	X	X	X						
L4	x		X	X		x	X	X		x			x	X
L5		X	X	X					x	X			X	X
														
S1		X	X	X					X	X	X	X	X	X
S2		X							X	X	X	X		

The innervation level is expressed as X (high) and x (low). X and x are weighted with *kij* = 1 and *kij* = 0.5, respectively in equation (2).

To evaluate the spatial characteristics of the MN activation patterns of each module, the peak activity segment height of the seven (from L1 to S2) lumbosacral segment was calculated in each module. In addition, to evaluate the temporal characteristics of the MN activation patterns of the module, peak timings of the temporal activations were analyzed by using circular statistics (Batschelet et al. 1981; Berens 2009). Then, the means of the peak timing of temporal activations across participants are calculated in each module type.

### Effects of normalization methods

Since the number of MNs among each spinal segment is different (Table 2) (Tomlinson and Irving 1977), the spatiotemporal activation patterns of MNs were normalized to the number of MNs in some previous studies (Ivanenko et al. 2013; La Scaleia et al. 2014). This normalization probably affects comparisons of peak activity segment height among each module type. To assess the effects of normalization, the activity of each spinal segment (*Sj*, in the equation (2)) was normalized based on the estimated number of MNs in the respective segment, according to a previous study (Ivanenko et al. 2013). Namely, the *Sj* was multiplied by the number of MNs in the respective segment and divided by the maximum number of MNs across seven segments (i.e., 12,765 in L3). From the normalized MNs activation patterns, the peak activity segment height of the seven lumbosacral segments (from L1 to S2) was calculated for each module.

**Table 2:** Mean number of motoneurons in each segment of the human spinal cord (13–40 years, 12 cases)

Segment height	Motoneuron number
L1	806
L2	5146
L3	12,765
L4	12,069
L5	12,674
S1	10,372
S2	4216

Data are adopted from Tomlinson and Irving (1977).

Since the activity of some muscles (e.g., triceps surae muscles) during air‐stepping was lower than when walking (Ivanenko et al. 2002; Gerasimenko et al. 2010), their relative activation differed from normal walking. Since the differences in the EMG amplitude affect module extraction by NMF, we assessed whether the results obtained in this study were just derived from the imbalance of EMG amplitude among muscles. Specifically, the EMG amplitude of each muscle was normalized to the maximum value for that muscle over the air‐stepping task. Using the normalized EMGs, motor modules were extracted, and then MNs activation patterns were reconstructed using the same methods mentioned above.

### Statistics

Differences in the peak activity segment height among each module type were compared using a Kruskal–Wallis test (nonparametric one‐way analysis of variance [ANOVA] test) with the Steel‐Dwass post hoc test (nonparametric Tukey's test). All the above statistical analyses were performed using EZR (Saitama Medical Center, Jichi Medical University, Japan) (Kanda 2013), which is a graphical user interface for R statistics (The R Foundation for Statistical Computing, version 2.13.0). In addition, differences of peak activation timings (i.e., mean angles of circular observations) among module types were tested by the Watson‐Williams test using a toolbox made by Berens (2009) for MATLAB (version R2016b, Mathworks, Natick, MA, USA). The p‐values were adjusted by Holm's correction for multiple comparisons using a custom‐written MATLAB script according to a previous paper (Holm 1979). Statistical significance was accepted at *P *<* *0.05.

## Results

### Kinematic data

Figure 1B shows a typical example of kinematic pattern of the one‐leg air stepping in a single participant. Figure 1C shows ensemble averages across participants of hip, knee, and ankle angles of the one‐leg air‐stepping. In the extension‐flexion cycle, the average terminal extension timing (i.e., peak timing of the hip extension angle) was 52.6 ± 1.2%. The temporal characteristics of these joint movements were generally similar to those of walking (Winter et al. 1974; Murray et al. 1984; Kadaba et al. 1990). Nevertheless, there were some differences between air‐stepping and walking as described below. In hip joint angle, the maximum flexion angle (83.3 ± 13.2°) was larger compared with that of walking (approximately 45°). The maximum flexion angle of the knee joint (115.4 ± 4.0°) was greater than that of walking (approximately 60°). Additionally, although a small knee flexion occurs after foot contact in level walking (corresponding to the early extension phase in air‐stepping), it was absent during air‐stepping. In ankle joint angle, the magnitude of the range of motion between air‐stepping (36.3 ± 16.5°) and level walking (approximately 30°) was similar, while the central angle in air‐stepping was shifted toward plantar flexion compared with that in level walking (39.2 ± 8.0° [planter flexion] in air‐stepping, approximately 5° [planter flexion] in level walking). Additionally, the ankle was continuously dorsiflexing during the stance phase and rapidly plantarflexed at the transition timing from the stance to swing phase in level walking; continuous plantar flexion was observed during the extension phase in air‐stepping.

### Motor modules extracted from EMGs

**Figure 3:**
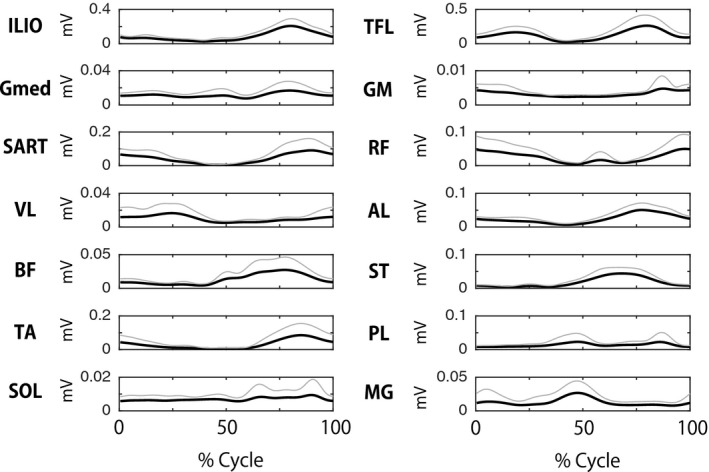
Muscle activation patterns during step cycles. Ensemble averaged activity patterns across participants (black lines) and their standard deviations (SD, gray lines) are shown.

**Figure 4:**
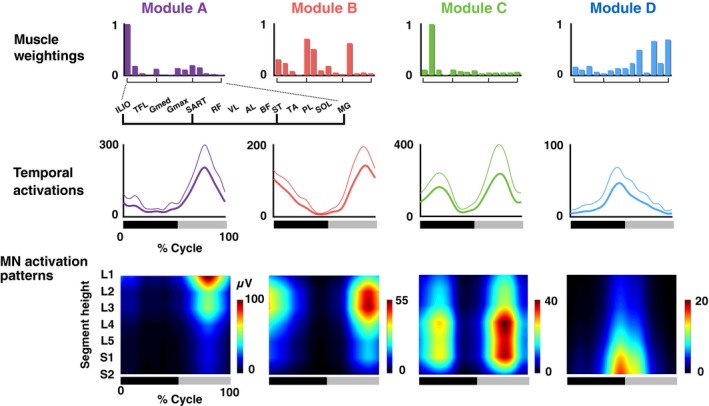
Muscle weightings, temporal activation patterns, and motoneuron (MN) activation patterns of extracted motor modules. Average muscle weightings across participants in each type of modules are shown. Each bar height represents the relative level of activation of each muscle within the muscle weighting components. Lines indicate the temporal pattern components of the modules. Average patterns across participants (thick lines) and their standard deviations (thin lines) are represented. Heat map indicates MN activity patterns generated by each module. The bars underneath denote the extension phase (black) and flexion phase (gray) of hip joint angle in a step cycle.

Figure 3 shows the EMG patterns generated during the one‐leg air‐stepping task. From the recorded EMGs (Fig. 3), 3.22 ± 0.83 motor modules were extracted from each participant. The extracted motor modules were grouped into four types by cluster analysis (Fig. 4, upper and middle rows). Module A was activated at the early‐mid flexion phase of hip joint angle and mainly recruited the ILIO. Module B was activated at the mid‐late flexion phase and mainly recruited the RF, SART, and TA. Module C was activated at twice (i.e., at the early‐mid extension and early‐mid flexion phases) and mainly recruited the TFL. Module D was activated at the last flexion‐initial extension phase and mainly recruited the ST, PL, and MG.

### Spatio‐temporal activation patterns of MNs generated from individual modules

**Figure 5:**
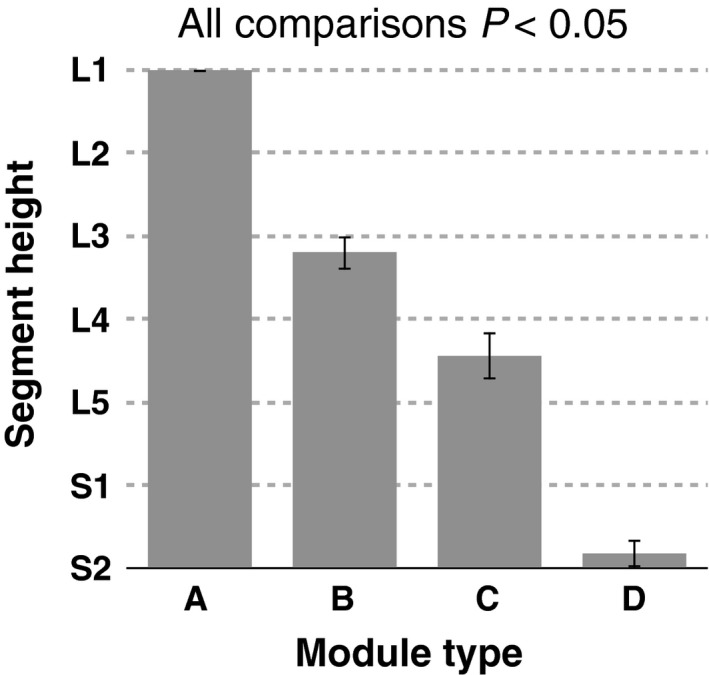
Group‐averaged peak activity segment height of the MN activity in the seven (from L1 to S2) lumbosacral segments for each motor module. Error bars indicate the standard errors.

**Figure 6:**
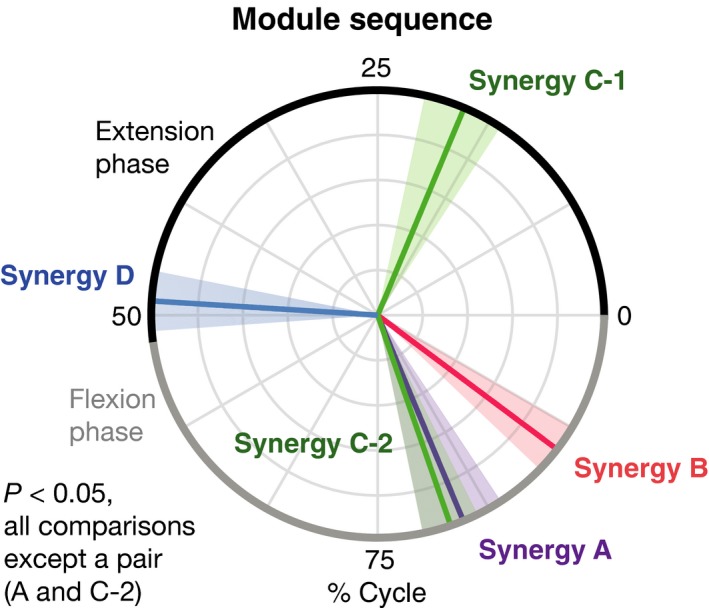
Motor module sequence in air stepping. Colored lines in the circular data indicate mean peak activation timings across participants. Translucent areas represent their standard errors.

The spatio‐temporal activation patterns of MNs were reconstructed from each motor module (Fig. 4, lower row). Based on visual inspection, each module activated the MN pools in specific segments at a specific timing point in the gait cycle. To quantify the differences in the activated segments, the peak activity segment heights of in the seven lumbosacral segments were calculated for each module (Fig. 5). Among the four module types, the segment heights of the CoA were high in the order of module A, B, C, and D (Kruskal–Wallis one‐way ANOVA: *P *<* *0.001; post hoc Steel–Dwass test: *P *<* *0.05). Additionally, using the circular statistic, we quantified the differences in peak timings of the temporal activations among modules (Fig. 6). As module C had two activation peaks as shown in Figure 4, we separately evaluated the first and second peaks as C1 and C2, respectively. The peak activation timings were different among the module types (*P *<* *0.05), except for a pair between modules A and C2. Thus, the sequence of the motor modules during the one‐leg air‐stepping was in the following order: C1, D, C2 + A, and B in the extension‐flexion cycle.

### Effects of normalization methods

**Figure 7:**
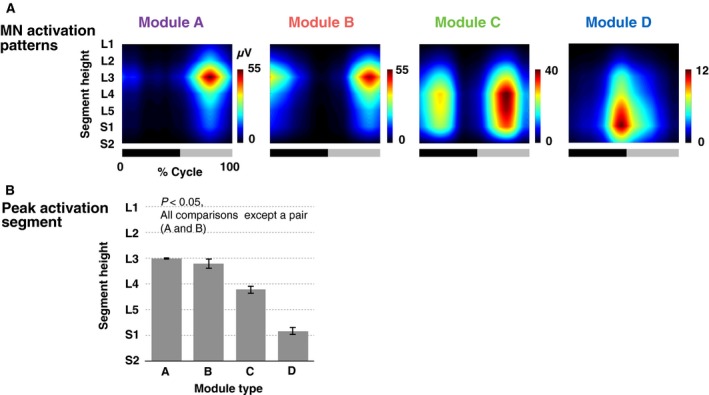
(A) MN activation patterns of motor modules normalized to MN numbers in each segment. The bars underneath denote the extension phase (black) and flexion phase (gray) of hip joint angle in a step cycle. (B) Group‐averaged peak activity segment height of the normalized MN activation patterns. Error bars indicate the standard errors.

Figure 7 shows the spatiotemporal MN activation patterns (Fig. 7A) and peak activity segment (Fig. 7B) for each module type after normalization to the MN number. Compared with the nonnormalized data (Fig. 4), the MN activation patterns were moved toward the center of lumbosacral enlargement. The normalization especially affected to the modules representing MN activations at the end of the lumbosacral enlargement (i.e., module A and D). Although there was no significant difference in the peak activity segment (Fig. 7B) between modules A and B, all the other comparisons showed significant differences (*P* < 0.05), which was the same as the nonnormalized data (Fig. 5).

**Figure 8:**
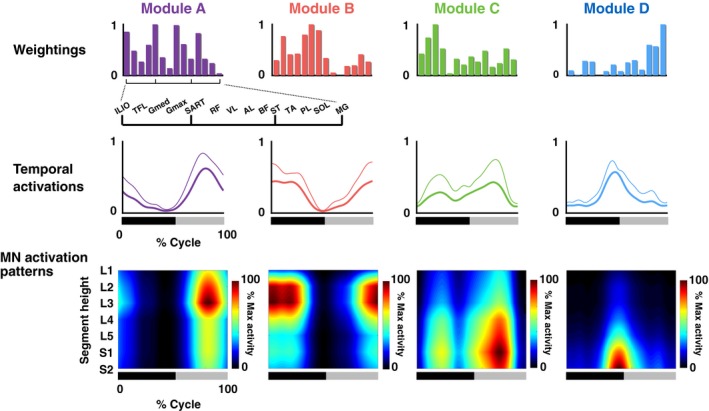
Muscle weightings, temporal activation patterns, and MN activation patterns of the motor modules extracted from amplitude‐normalized EMGs. Average muscle weightings across participants in each type of modules are shown. Each bar height represents the relative level of activation of each muscle within the muscle weighting components. Lines indicate the temporal pattern components of the modules. Average patterns across participants (thick lines) and their standard deviations (thin lines) are presented. Heat map indicates MN activity patterns generated by each module. The bars underneath denote the extension phase (black) and flexion phase (gray) of hip joint angle in a step cycle.

We extracted modules from amplitude normalized EMGs. As a result, 3.80 ± 0.75 (mean ± SD) motor modules were extracted from each participant, and the modules were grouped into four types by the cluster analysis, which was the same as in the nonnormalized data. Figure 8 shows the muscle weightings, temporal activations, and MN activation patterns of the motor modules extracted from the amplitude normalized EMGs. Compared with nonnormalized data (Fig. 4), the muscle weightings (upper row) of several muscles were highly weighted in modules A–C. The temporal patterns (middle row) represented quite similar patterns. Regarding the MN activation patterns of the modules (Fig. 8), the activated segments in module A shifted from L1 (nonnormalized data, Fig. 4) to L2–L3 (normalized data).

## Discussion

In this study, four different types (module A–D) of motor modules were extracted from muscle activity during air‐stepping. Among the module types, the peak activity segment height in the lumbosacral enlargement was high in the order of module A  >  B  >  C  >  D. In addition, the activation sequence of motor modules during the one‐leg air‐stepping was in the following order: C1, D, C2 + A, and B in the extension‐flexion cycle. The order can be sorted as “C2 + A‐B‐C1‐D” in the flexion‐extension cycle. The “A‐B‐C1‐D” sequence corresponded to the order of the activated segment heights, i.e., “A  >  B  >  C  >  D”. Comparing the activation sequence with the topography of the motor modules in the spinal cord suggests the existence of a rostrocaudally traveling wave of activation in human locomotor spinal circuits.

### Comparisons of bipedal walking and air‐stepping

Based on the myotomal charts (Kendall et al. 1993), a previous study (Ivanenko et al. 2006) examined the activation of lumbosacral MNs from EMGs during walking in humans. Although Ivanenko et al. (2006) observed rostrocaudal wave‐like activation, the activation was shifted rostrally toward the upper lumbar segments around the time of the foot contact timing. Consequently, the MN activations showed rostrocaudal movements with two cycles in a gait cycle. The motor module mainly activated the knee extensor muscles and was considered to be related to the loading response at foot contact.

Therefore, this study aimed to examine the activation of the lumbosacral spinal cord in a stepping movement, without foot‐contact interactions, using an air‐stepping task (Ivanenko et al. 2002, 2007). From the muscle activity during air‐stepping, four types of motor modules were extracted (Fig. 4). As expected, the motor module for the loading response at foot contact (corresponding to the initial extension phase) was not extracted. The four extracted motor modules in this study were similar to those extracted in previous studies examining motor modules during human locomotion (Cappellini et al. 2006; Neptune et al. 2009; Chvatal and Ting 2012; Yokoyama et al. 2016).

Module A activated at the time of early‐mid flexion phase of the hip joint, and it mainly recruited the ILIO (Fig. 4). This module acts to accelerate the leg forward in the early swing (Neptune et al. 2009) and showed the highest amplitude activation among the four module types during air‐stepping (Fig. 4). This characteristic is not seen in walking and would be caused by the greater hip flexion during air‐stepping as mentioned in the Results. Module B activated at the time of mid‐late flexion phase, and mainly recruited the RF, SART, and TA. This module was related to toe clearance and leg acceleration in the mid‐late swing phase (Neptune et al. 2009). Module C activated at twice (i.e., during the early‐mid extension and early‐mid flexion phases), and mainly recruited the TFL. During walking, this module activated during walking at one peak timing, i.e., at the early stance (corresponding to early‐mid extension), to stabilize the pelvis (Yokoyama et al. 2016). The TFL, major muscle of the module C, was almost inactive at the swing phase of normal walking (Yokoyama et al. 2016). However, it would act at the initial swing for additional pelvic stability and supplementary thigh acceleration under a large hip flexion angle during the air‐stepping as demonstrated in up‐ramp and up‐stair walking (Gottschall et al. 2012). Module D activated at the last extension‐initial flexion phase of hip joint angle, and it mainly recruited the ST, PL, and MG. During walking, this module plays a role in plantar flexion/body support and propulsion at late stance (Chvatal and Ting 2012).

### Topography and temporal sequences of MN activations in air‐stepping

The difference of the peak activity segment among the modules A  >  B  >  C  >  D (Fig. 5) corresponded with the with specificity of encoding locations of motor modules in the spinal cord of frogs (Saltiel et al. 2015) and mice (Caggiano et al. 2016). Regarding the temporal sequence of modules, “C2 + A‐B‐C1‐D” (Fig. 6), although the peak timings of A, B, C1, and D were consistent with those observed in level walking (Cappellini et al. 2006; Neptune et al. 2009; Chvatal and Ting 2012; Yokoyama et al. 2016), C2 is not seen in level walking. If evaluating only the module activations related to level walking (i.e., A, B, C1, D), the order of the activated segment height, “A  >  B  >  C  >  D”, and the activation sequence, “A‐B‐C1‐D”, indicate that motor modules were sequentially activated from the anatomically upper module to the lower module. The locomotor CPG is considered to consist of temporal structures and pattern formation networks, and the motor modules may underlie the pattern formation networks (McCrea and Rybak 2008). Therefore, the sequential rostrocaudal activation of the motor modules suggests the possibility that rostrocaudally traveling waves of activation exist in human locomotor spinal circuits as a temporal structure to activate motor modules in proper sequences.

Although the topography and temporal order was corresponded in flection‐extension cycle, the temporal order depended on the onset of the gait cycle. Regarding onset of gait cycle, it has been well known that swing onset is regulated by two types of sensory information force afferents of the ankle extensor muscles (Duysens and Pearson 1980; Whelan et al. 1995) and position afferents from the hip flexor muscles (Grillner and Rossignol 1978; Kriellaars et al. 1994; Hiebert et al. 1996). Therefore, we consider that the gait cycle is initiated from onset of hip flexion from the viewpoint of neural control. Additionally, our results are consistent with the traveling wave of activations that occurred during the swing‐stance cycle in flogs (Saltiel et al. 2015).

### Traveling wave of activations in the spinal cord in nonhuman vertebrates

The rostrocaudal distribution organization of CPG is supported by theoretical studies (Kaske et al. 2003; Ijspeert et al. 2007) and experimental studies on EMG activity, spinal MNs, and interneurons among many vertebrates including Lamprey (Wallén and Williams 1984), fish (Grillner 1974), tadpoles (Roberts et al. 1998), frogs (Saltiel et al. 2015), rodents (Cazalets 2005), and cats (Cuellar et al. 2009; Pérez et al. 2009). For example, Cuellar et al. (2009) demonstrated a traveling wave of cord dorsum potentials by interneuronal recordings during the fictive cat scratch, indicating the rostrocaudal interneuronal activation in the mammalian spinal locomotor circuits. In rodents, rostrocaudal propagation of MN activation was optically imaged (Bonnot et al. 2002; O'donovan et al. 2008), and the traveling waves were also electrophysiologically recorded from the ventral roots (Cazalets 2005). In addition, a study by Saltiel et al. (2015) demonstrated a traveling wave in the spinal cord in frog locomotion based on motor module temporal sequences and topography in the spinal cord, which is similar to our results.

Regarding the neural origin of the traveling wave of activation in the spinal cord, rostrocaudal propagation of spontaneous electrical activity of dorsal horn neurons in the layers III–VI (i.e., cord dorsum potentials) is considered as a temporal structure of CPG networks (Cuellar et al. 2009; Pérez et al. 2009; Kato et al. 2013). Namely, the MN clusters corresponding to each motor modules are probably sequentially recruited from rostral regions to caudal regions by the rostrocaudal propagation of the cord dorsum potentials. In rodents, neurons in the layer III–V have interesting anatomical features that may play a role in propagating the traveling wave (Schneider 1992). The neurons have an axon running in the rostrocaudal direction, with perpendicular collateral branches that are intermittently spaced apart. If this type of neurons is also present in humans, then it is possible that cord dorsum potentials of the neurons are an underlying mechanism of the traveling wave of activations in the spinal cord, which control locomotion.

### Possibility of traveling waves in human locomotor circuits

To date, the traveling wave of activation has not been confirmed in human locomotion. Nevertheless, it has been suggested that CPGs in legged vertebrates have evolved from common ancestral circuit for undulatory locomotor behaviors, such as fish and lamprey (Grillner and Jessell 2009). In addition, EMG‐based studies strongly suggested that the motor modules of humans and those of other legged vertebrates share similar circuitries (Dominici et al. 2011; Yokoyama et al. 2017). Based on the commonality of spinal locomotor circuits, it is conceivable that traveling waves are the neural mechanisms underlying the motor module sequence and the topography of the motor modules presented in this study.

In human walking, in addition to the MN activations, which are thought to be related to the traveling wave, MNs in the rostral lumbosacral enlargement are activated around foot contact (Ivanenko et al. 2004). The additional MN activation mainly recruits two modules (Ivanenko et al. 2004; Yokoyama et al. 2017), which have essential biomechanical functions to support and decelerate the body during the loading response (Ivanenko et al. 2004; Neptune et al. 2009; Chvatal and Ting 2012). One of the modules, which mainly recruits the quadriceps, may be recruited by loading afferent inputs, because the activation of the quadriceps decrease depending on the amount of relief under bodyweight supported walking (Ivanenko et al. 2002; Klarner et al. 2010). Another module, which mainly recruits the TA, may be mainly recruited by cortical commands, because the TA has significant connectivity with motor cortex prior to foot contact (Petersen et al. 2012) and the amplitude is not correlated with the amount of body weight relief (Ivanenko et al. 2002; Klarner et al. 2010). Thus, combined motor module activations derived from rostrocaudally traveling waves, sensory inputs and cortical commands may contribute to the generation of coordinated muscle activity during walking.

### Effect of different normalization methods

Some previous studies normalized the spatiotemporal activation patterns of MNs based on the number of MNs in each segment (Ivanenko et al. 2013; La Scaleia et al. 2014). We examined the effects of this normalization to the peak activity segment (Fig. 7). The peak activity segment was significantly different among motor module types in nonnormalized data (Fig. 4), while the significant difference between module A and B was lost after the normalization (Fig. 7). This could be attributed to the fact that this normalization mainly affected on the end segments of the lumbosacral enlargement (L1, L2 and S2), which contained considerably lower numbers of MNs than other segments (Table 2). Although there was no significant difference of the peak activity segment between module A and B, the fact remains that activation locations of both modules were upper lumber regions at initial flexion phase. Thus, since MNs activated from rostral region to caudal region during the activation sequence, “A‐B‐C1‐D”, the normalized results still do not refute the existence of a traveling wave of activation in human spinal circuits.

We also examined the effects of EMG amplitude normalization on the motor module extraction and MN activity reconstruction (Fig. 8). Compared to the nonnormalized data (Fig. 4), although the temporal patterns were similar, the muscle weightings were different in modules A–C. Although there was a slight shift in the activated segments in module A, from L1 (nonnormalized data) to L2‐L3 (normalized data), overall, the MN activation patterns of the other modules (Fig. 8) were qualitatively similar to that of the nonnormalized data (Fig. 4). Even after the normalization, the activation location of module A was the upper lumbar region, indicating that the MNs were activated from rostral region to caudal region during the module sequence. Therefore, the normalized results also do not refute the hypothesis that traveling waves of activation exist in human spinal circuits.

### Methodological considerations

There are several limitations in this study that must be noted. We demonstrated the possible existence of the traveling wave in human spinal circuits, using voluntary leg movement. Human locomotor muscle activity is suggested to be generated by the spinal CPGs (Duysens and Van de Crommert 1998). However, because the cortex controls walking, the possibility that the sequential activation of motor modules in the rostrocaudal direction are independently activated by each corresponding voluntary descending drive cannot be ruled out in humans (Petersen et al. 2012; Artoni et al. 2017). To examine the neural origin of traveling wave, studies on individuals with SCI would provide more direct evidence with eliminating the effects of descending drives. A recent study on SCI showed that the epidural electrical stimulation to the lumbar spinal cord could elicit coordinated rhythmic activities of multiple lower leg muscles innervated from a wide range of lumbosacral segments (Danner et al. 2015). Consistent with our results, this study also showed that four motor modules acting at different peak timings were extracted from the coordinated muscle activities generated by spinal circuits (Danner et al. 2015). Thus, the examination of the topography of the motor modules extracted from patients with SCI would provide more direct evidence of the traveling wave in the human spinal cord.

Another limitation of our study is that we examined locomotor networks, using unilateral leg movements. Although locomotor CPGs have bilateral couplings for left‐right coordination (Butt and Kiehn 2003; Maclellan et al. 2014), unilateral coupling for controlling unilateral leg has been also shown in electrophysiological (Hägglund et al. 2013) and behavioral (Yang et al. 2004; Choi and Bastian 2007) studies. Therefore, the spinal locomotor CPGs consist of bilateral and unilateral components (Kiehn 2016). The traveling wave of activation in the spinal cord has been shown as a mechanism for the temporal regulation in the unilateral component of the CPGs (Cuellar et al. 2009; Saltiel et al. 2015). Thus, the results in this study would reflect the characteristics of the unilateral component of the locomotor circuits in the human spinal cord.

Another important methodological concern is the potential issue of EMG cross talk. The issue of cross talk is particularly applicable to the EMG signal of the ILIO muscle in this study. Although the ILIO is one of the deep muscles, a recent MRI study demonstrated that the superficial region of this muscle in the femoral triangle immediately under the inguinal region is adequately large for surface EMG recordings (Jiroumaru et al. 2014). In this study, to adequately attach the electrodes on the superficial region, we carefully checked the location by manual palpation (Muscolino 2008) and cross‐talk tests (Criswell and Cram 2011). Nevertheless, we cannot completely rule out the possibility that the EMG signal of the ILIO was still contaminated by cross talk from adjacent muscles, such as the sartorius muscle (SA) and the internal oblique muscle (IO). In this study, as the ILIO was mainly recruited by module A, which was activated from upper lumber segments (Fig. 4). Since the SA and the IO are innervated from upper lumber segments and thoracic segments (SA: L2–L3, IO: T7‐L1) (Kendall et al. 1993), the module A would be activated from more upper region if the EMG signal of the ILIO was contaminated by the SA and the IO activity. Thus, we believe that the cross talk in the EMG signal of the ILIO had little effect on the rostrocaudally traveling waves of activation in this study.

## Summary

We examined whether the traveling wave of activation existed in the human spinal circuits by extracting motor modules and reconstructing the MNs activity of the modules during air‐stepping. Our results suggest the possibility that neural mechanisms of rostrocaudally traveling waves of activation are conserved in human spinal locomotor circuits. This neural mechanism would take advantage of activating motor modules in proper sequences to generate locomotor muscle activity in humans. The results would also provide novel information on the spatial arrangement of MNs for movement control, which has been studied over many years (Romanes 1964; Jessell et al. 2011). In addition, the commonality about the traveling waves of activation between humans and other vertebrates supports the hypothesis that fundamental locomotor networks are conserved across phylogenetic and morphological differences in vertebrates (Grillner and Jessell 2009; Dominici et al. 2011). Therefore, we believe that the results of this study advance our understanding of human locomotor control mechanisms, and provide important insights into the evolution of locomotor networks in vertebrates.

## Conflict of Interest

We have no competing interests.
